# Effectiveness of adjunctive herbal medicine in the management of post-stroke thalamic pain: a systematic review and meta-analysis

**DOI:** 10.3389/fneur.2026.1717558

**Published:** 2026-07-31

**Authors:** Junying Zhai, Tianwei Zhang, Xuran Zhang, Ling Cheng, Lifang Ma, Xiao Han, Zhongjian Tan, Fang Wang, Xinglu Dong, Li Zhou

**Affiliations:** 1 Dongzhimen Hospital, Beijing University of Chinese Medicine, Beijing, China; 2 Beijing University of Chinese Medicine, Beijing, China; 3 Pain Department, Eye Hospital, China Academy of Chinese Medical Sciences, Beijing, China

**Keywords:** alternative medicine, central pain, herbal medicine, meta-analysis, post-stroke thalamic pain, stroke

## Abstract

**Background:**

Post-stroke thalamic pain (PSTP) represents one of the most challenging types of refractory central neuropathic pain. The application of herbal medicine alongside Western medicine (WM) has been proposed as a potential therapeutic option for managing PSTP. Nevertheless, its comprehensive effectiveness and safety have yet to be conclusively established.

**Objective:**

This study aimed to systematically evaluate the efficacy and safety of herbal medicine combined with WM for the management of PSTP.

**Methods:**

Eight electronic databases were searched from inception to August 2025 to retrieve randomized controlled trials (RCTs) evaluating combined herbal medicine and WM against WM monotherapy in the management of PSTP. Outcomes included pain intensity measures (VAS, PPI, PRI), anxiety and depression scales (HAMA, SAS, HAMD and SDS), overall treatment effectiveness and adverse events. Sensitivity and subgroup analyses were performed to explore potential sources of heterogeneity. The GRADE framework was used to appraise the strength of evidence for specific results. Trial sequential analysis was carried out with TSA software.

**Results:**

The systematic review encompassed 16 trials, incorporating 1,122 participants. The pooled analysis showed that herbal medicine combined with WM was associated with a higher total effective rate than WM alone (RR = 1.38, 95% CI: 1.28–1.49, *p* < 0.00001). Compared with WM alone, herbal medicine combined with WM significantly reduced VAS score (MD = −1.50, 95% CI: −1.84 – -1.16, *p* < 0.00001), PPI score (MD = −1.39, 95% CI: −1.55 – -1.22, *p* < 0.00001), and PRI score (MD = −2.46, 95% CI: −3.24 – -1.69, *p* < 0.00001). Combination therapy also effectively reduced anxiety (HAMA: MD = −3.10, 95% CI: −3.53 – -2.67, *p* < 0.00001; SAS: MD = −6.97, 95% CI: −7.49 – -6.46, *p* < 0.00001) and depression (HAMD: MD = −2.26, 95% CI: −2.72 – -1.80, *p* < 0.00001; SDS: MD = −7.15, 95% CI: −7.66 – -6.65, *p* < 0.00001). Regarding safety, herbal medicine combined with WM was not associated with an increased incidence of adverse events compared with WM alone (RR = 0.95, 95% CI: 0.55–1.64, *p* = 0.86). The certainty of evidence assessed using GRADE ranged from moderate to very low, and TSA provided additional statistical support for the potential benefit of the combined therapy.

**Conclusion:**

Evidence from this systematic review indicates that adjunctive herbal medicine combined with WM may have potential benefits in reducing pain, anxiety, and depression, and improving clinical response in patients with PSTP. However, these findings should be interpreted with caution due to methodological limitations and the exclusive inclusion of studies from China. Further rigorous, multicenter, and multinational trials are needed to confirm these findings.

**Systematic review registration:**

The systematic review was registered in PROSPERO (registration number: CRD42024494796).

## 1 Introduction

Central post-stroke pain (CPSP) is a disabling complication of cerebrovascular injury, often manifesting as chronic or episodic pain associated with sensory abnormalities (1–3). Post-stroke thalamic pain (PSTP) represents one of the most severe phenotypes of CPSP and is characterized by contralateral hyperalgesia and dysesthesia (4).

It has been observed that patients with PSTP present increased mechanical detection thresholds as well as decreased mechanical pain detection thresholds relative to individuals with a history of thalamic stroke without pain (5). It usually progresses over time, severely impairing quality of life and associated with psychological comorbidities (6). A vicious cycle may develop in which pain interacts with anxiety and depression, further amplifying pain perception and worsening neurological symptoms (7).

Although the exact pathogenesis remains unclear, current evidence suggests that vascular lesions disrupt the spinothalamic pathway and thalamocortical circuits, leading to somatosensory dysfunction, neuronal hyperexcitability, and central sensitization (8). Additional mechanisms, such as microglial activation and inflammatory responses, may further contribute to maladaptive neural plasticity (4). Despite these insights, the complex and multifactorial nature of PSTP has hindered the development of effective and targeted treatments. The therapeutic mainstay for PSTP, per current guidelines, includes a range of pharmacotherapies—namely antidepressants, anticonvulsants, and opioids, in addition to neuromodulation techniques (9), but these approaches often fail to achieve satisfactory outcomes (10). Herbal medicine is attracting growing interest owing to its well-established safety and extensive use in clinical settings.

From the perspective of traditional Chinese medicine, PSTP is classified as “stroke-related impediment” (11), with pathogenesis centered on qi-blood stagnation and meridian obstruction. Herbal prescriptions that promote blood circulation, unblock meridians, or tonify qi and nourish blood have long been used to relieve pain (12). Increasing evidence indicates that active compounds in Chinese herbs, including glycosides, flavonoids, phenols, and terpenoids, possess neuroprotective and restorative effects (13, 14). Recent clinical studies suggest that integrative therapy combining herbal medicine and Western medicine (WM) yields superior efficacy compared with pharmacotherapy alone (15), although systematic evidence remains insufficient. Therefore, this study systematically evaluated the efficacy and safety of herbal medicine combined with WM for PSTP, aiming to inform clinical practice and future research on integrative treatment strategies.

## 2 Data and methods

This evaluation was posted on PROSPERO (registration number: CRD42024494796).

### 2.1 Search strategy

Our search strategy encompassed several major electronic databases. These included international databases such as PubMed, EMBASE, Web of Science, and the Cochrane Library, along with widely used Chinese databases like China National Knowledge Infrastructure (CNKI), Wanfang, VIP, and China Biomedical Database (CBM). Search period extended from their initial entries through August 14, 2025. Keywords employed in the search included: post-stroke thalamic pain, thalamic pain, central post-stroke pain, herbal medicine, traditional Chinese medicines, Chinese herbal medicine, Chinese and Western medicine, Chinese patent medicine, and randomized controlled trial. For each database, the search strategy was refined to match its specific features, with an example provided for PubMed in Table 1.

**Table 1:** The search strategy of PubMed.

Number	Search terms
#1	post-stroke thalamic pain[Title/Abstract]
#2	central post-stroke pain[Title/Abstract] OR CPSP[Title/Abstract]
#3	#1 OR #2
#4	Thalamic Diseases[Mesh]
#5	Disease, Thalamic[Title/Abstract] OR Diseases, Thalamic[Title/Abstract] OR Thalamic Disease[Title/Abstract] OR Dejerine-Roussy Syndrome[Title/Abstract] OR Dejerine Roussy Syndrome[Title/Abstract] OR Syndrome, Dejerine-Roussy[Title/Abstract] OR Thalamic Syndrome[Title/Abstract] OR Syndrome, Thalamic[Title/Abstract] OR Syndromes, Thalamic[Title/Abstract] OR Thalamic Syndromes[Title/Abstract]
#6	#4 OR #5
#7	#3 OR #6
#8	randomized controlled trial [Publication Type] OR randomized [Title/Abstract] OR placebo [Title/Abstract]
#9	#7 AND #8

### 2.2 Inclusion criteria

- Study Design: Only randomized controlled trials (RCTs), conducted either in China or abroad and reported in Chinese or English, were considered.
- Participants: Eligible individuals were patients clinically diagnosed with PSTP, regardless of race, nationality, or disease duration. Diagnosis of stroke required CT or MRI evidence of vascular lesions within the thalamus. Participants experienced spontaneous pain in the limb contralateral to the lesion, frequently accompanied by sensory abnormalities.
- Interventions: In the control group, participants received conventional WM therapy alone, whereas those in the intervention group received herbal medicine in addition to WM. Within each trial, background therapies were consistent across study arms. No limitations were placed on treatment duration.
- Outcomes: Studies were required to assess at least one relevant endpoint, including overall clinical effectiveness, pain intensity, psychological distress, or adverse events.

Total effective rate was extracted as reported in the original studies. It was defined based on the criteria used in each individual trial, typically categorizing patients as cured, markedly improved, improved, or non-responders. The total effective rate was analyzed as the proportion of patients who achieved a clinical response.

Pain intensity was assessed using the Visual Analog Scale (VAS), Pain Rating Index (PRI), or Present Pain Intensity (PPI) scale. Psychological distress was evaluated using the Self-Rating Anxiety Scale (SAS), Hamilton Anxiety Scale (HAMA), Self-Rating Depression Scale (SDS), or Hamilton Depression Scale (HAMD). Adverse events were also recorded.

### 2.3 Exclusion criteria

- Preclinical Evidence: Publications limited to animal experiments or laboratory-based studies were excluded.
- Non-randomized Research: Articles other than RCTs: including reviews, meta-analyses, case reports, expert commentaries, and theoretical discussions were not eligible.
- Redundant Reports: Duplicate publications, overlapping datasets, or suspected repeated analyses were excluded.
- Unavailable Data: Studies without accessible or complete full texts were not considered.

### 2.4 Literature screening and data extraction

Early-stage screening focused on titles and abstracts was performed by two investigators (Xuran Zhang and Ling Cheng) in an independent manner. Records that clearly did not meet the inclusion criteria were excluded. Full texts of potentially eligible studies were further assessed to determine final inclusion. The findings of the included research were cross-checked by the two investigators. Any disagreements or concerns about their inclusion were resolved through discussion with a third investigator. For the included literature, the data extraction process captured key study characteristics, including authorship, publication date, sample size, demographic characteristics (e.g., age), interventions, trial duration, and any reported adverse effects.

### 2.5 Evaluation of literature quality

Both evaluators independently screened and appraised the methodological rigor and the extent of bias across the included trials. Disagreements underwent initial discussion between evaluators to reach consensus on inclusion. Any persistent disagreements were arbitrated by a senior researcher to arrive at a final conclusion. The Revman (5.4) criteria were utilized to evaluate the listed studies’ methodological quality in terms of possible bias. We appraised the risk of bias by examining several critical aspects: firstly, the methods for generating the random allocation sequence and ensuring its concealment until assignment were scrutinized for potential selection bias. Secondly, we considered whether blinding was applied to participants, treatment providers, and outcome assessors. Further appraisals focused on the completeness of the outcome data, whether all pre-specified outcomes were reported, and the likelihood of any other bias influencing the results.

### 2.6 Statistical analyses

The statistical computations were conducted using RevMan (5.4) along with Stata version 16. For dichotomous variables, relative risk (RR) was selected as the effect measure. Mean difference (MD) was utilized for continuous outcomes measured on the same scale. A 95% confidence interval (CI) accompanied each effect size. The Cochrane Q test and *I*^2^ statistics were used to assess heterogeneity across studies. A common-effect model was used when *p* > 0.1 and I^2^ < 50%, whereas a random-effects model was adopted otherwise. When necessary, subgroup analyses were performed to identify possible causes of heterogeneity. Differences reaching statistical significance were noted if the *p* value, obtained from the Z-test, was below 0.05. Publication bias was assessed using funnel plots and Egger’s test.

### 2.7 Evidence quality evaluation

Utilizing Grading of Recommendations Assessment, Development and Evaluation (GRADE) profiler, strength of the evidence of the included trials was evaluated. An independent assessment of the evidence certainty was conducted by two reviewers (Junying Zhai and Tianwei Zhang) in accordance with the GRADE criteria. A consensus process was employed to resolve any discrepancies between their evaluations. Primary criteria included publication bias, inconsistency, indirectness, imprecision, and study limitations. Following GRADE framework, the evidence was stratified into one of four certainty levels: high, moderate, low, or very low.

### 2.8 Trial sequential analysis

To reduce the risk of random errors, we performed trial sequential analysis to assess the reliability of the findings with TSA software (version 0.9.5.10). Boundaries were observed using trial sequential monitoring and required information size (RIS) calculations. These aimed to achieve a 25% relative risk reduction, a two-sided alpha level of 0.05 and a beta level of 0.10. Low-bias studies were included to assess control group incidence, mean difference, and variance.

## 3 Results

### 3.1 Study selection and characteristics

**Figure 1:**
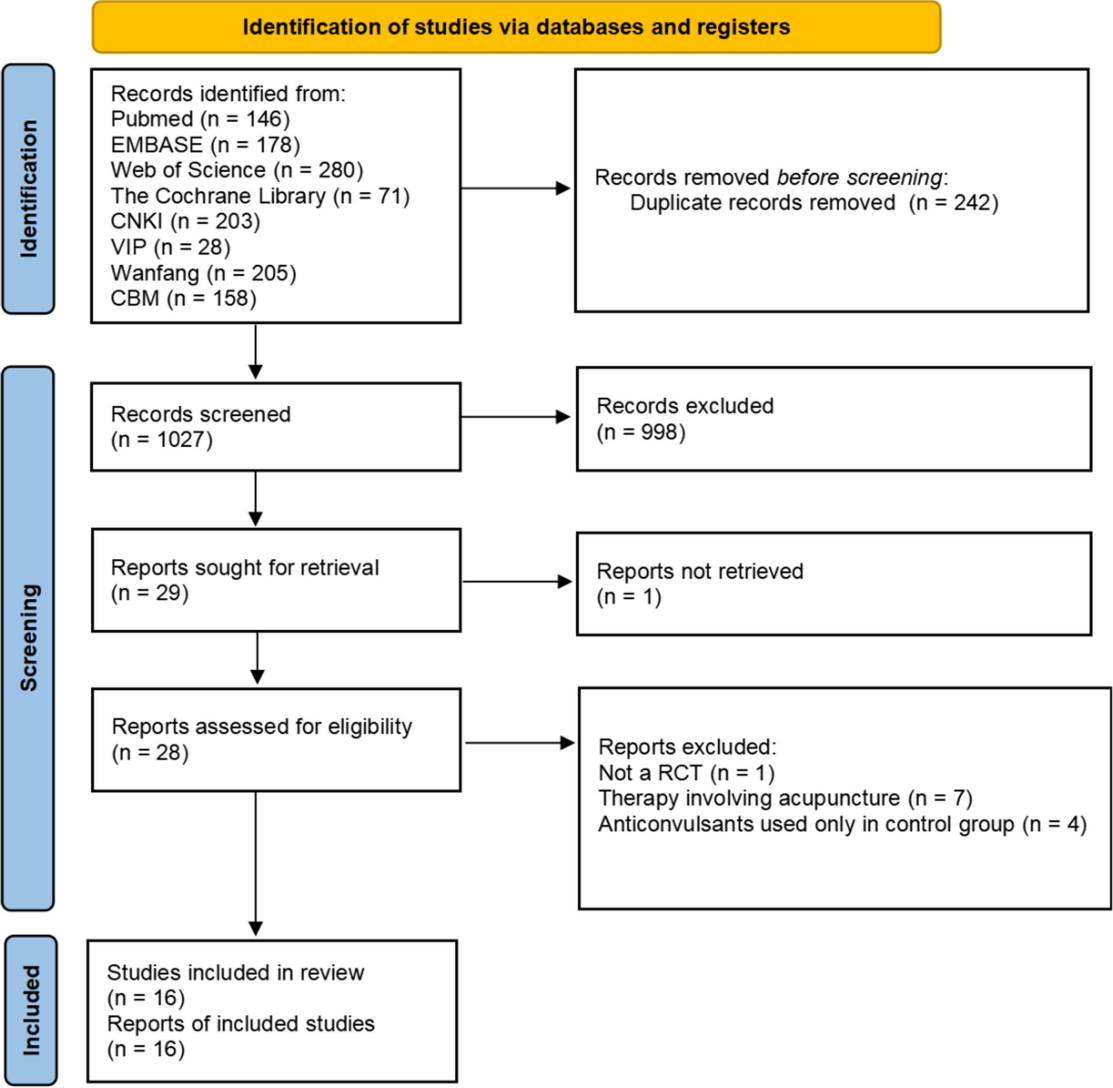
PRISMA flow diagram.

Based on our search approach, 1,269 articles were initially found overall. These records were managed using EndNote X9.2, resulting in 1,027 unique articles after duplicates were removed. A total of 998 studies were removed during the first-round screening, and the remaining 29 were retrieved for full-text review. One record could not be obtained in full text, leaving 28 articles for in-depth review. Full-text screening excluded one non-randomized study, seven trials involving acupuncture, and four studies in which anticonvulsants were used only in the control group. Finally, 16 eligible articles were incorporated in the analysis (16–31). Figure 1 provides an overview of the literature screening process and corresponding outcomes.

The analysis comprised 16 RCTs with 1,122 patients confirmed to have PSTP. Of these, 564 patients were in the experimental group receiving combined herbal medicine and WM, while 558 patients were in the control group receiving only WM. Intervention duration across all studies ranged from 14 to 56 days. Random sampling methods were explicitly stated in 13 studies. Individual RCTs varied in sample size, with the largest study including 120 cases and the smallest including 38 instances. Table 2 provides a comprehensive summary of study characteristics evaluated within this meta-analysis.

**Table 2:** Summary of characteristics of the included studies.

Studies	Sample size (exp/con)	Sex (M/F) (exp vs con)	Age (exp vs con)	Intervention measures (experimental group vs control group)	Duration of intervention (follow-up)	Outcomes
Xu (19)	30/28	—	—	modified Huangqi Guizhi Wuwu Decoction + carbamazepine	carbamazepine	4 wks (Not done)	①②
Gong and Chen (20)	33/32	18/15 vs. 17/15	61.27 ± 8.11 vs. 61.34 ± 8.02	Jiawei Shentong Zhuyu Decoction + Flupentixol and Melitracen Tablet	Flupentixol and Melitracen Tablet	4 wks (Not done)	①②
Guo et al. (21)	38/38	21/17 vs. 23/15	62.23 ± 7.90 vs. 60.53 ± 8.90	Shaoyao Gancao Decoction + gabapentin	gabapentin	8 wks (Not done)	①②③④
Zhang (18)	32/32	18/14 vs. 16/16	60.81 ± 8.36 vs. 61.75 ± 8.43	Xiao Huoluo Dan + carbamazepine	carbamazepine	4 wks (Not done)	①②
Bi et al. (22)	30/30	17/13 vs. 16/14	60.93 ± 8.05 vs. 59.67 ± 8.53	modified Shentong Zhuyu Decoction + neurotropin	neurotropin	4 wks (Not done)	①②
Qu (23)	33/32	17/16 vs. 17/15	62.47 ± 8.11 vs. 61.34 ± 8.02	Huoxue Zhuyu Decoction + Flupentixol and Melitracen Tablet	Flupentixol and Melitracen Tablet	4 wks (Not done)	①②
Tang et al. (24)	31/32	14/18 vs. 17/15	63.29 ± 7.46 vs. 63.68 ± 7.19	Buyang Huanwu Decoction + gabapentin	gabapentin	4 wks (Not done)	①②③
Shen (17)	29/30	17/13 vs. 18/11	60.23 ± 5.96 vs. 60.76 ± 6.02	MuDan Granule + carbamazepine	carbamazepine	8 wks (Not done)	①
Zeng and Wang (25)	30/30	17/13 vs. 16/14	66.4 ± 5.2 vs. 66.2 ± 5.8	Naodesheng Pill + gabapentin	gabapentin	4 wks (Not done)	①②③④⑤⑦
Liang and Ding (26)	60/60	29/31 vs. 26/34	50.30 ± 5.16 vs. 49.32 ± 5.1	modified Shentong Zhuyu Decoction + neurotropin	neurotropin	4 wks (Not done)	①②③④
Liu (27)	21/21	12/9 vs. 10/11	63.76 ± 7.02 vs. 63.54 ± 7.33	Xingnao Zhitong Decoction + fluoxetine	fluoxetine	2 wks (Not done)	①②③④⑥⑧
Li et al. (28)	19/19	10/9 vs. 6/13	53.58 ± 6.65 vs. 54.47 ± 8.28	Zhongfeng Huichun Tablet + gabapentin	gabapentin	4 wks (Not done)	①②
Dai and Qu (29)	49/44	27/22 vs. 24/20	62.4 ± 7.9 vs. 61.2 ± 8.4	modified Shentong Zhuyu Decoction + pregabalin	pregabalin	4 wks	①②⑥⑧
Li (16)	30/30	16/14 vs. 21/9	58.03 ± 5.43 vs. 57.10 ± 6.95	Daqinjiao Decoction + conventional treatment	conventional treatment	4 wks (Not done)	⑦
Peng et al. (30)	49/50	26/25 vs. 27/24	58.04 ± 13.20 vs. 59.31 ± 12.47	Yiqi Quzhuo Decoction + amitriptyline + flupirtine maleate	amitriptyline + flupirtine maleate	8 wks (Not done)	①②③④
Zhang et al. (31)	50/50	26/24 vs. 25/25	74.32 ± 4.35 vs. 74.54 ± 4.36	Xingnao Zhitong Decoction + fluoxetine	fluoxetine	2 wks (Not done)	①②⑤⑦

Outcomes: ①: total effective rate; ②: visual analog scale (VAS); ③: pain rating index (PRI); ④: present pain intensity (PPI); ⑤: self-rating anxiety scale (SAS); ⑥: Hamilton anxiety scale (HAMA); ⑦: self-depression scale (SDS); ⑧: Hamilton depression scale (HAMD).

WM treatments administered in the control group consisted of carbamazepine, flupentixol melitracen tablets, gabapentin, neurotropin, fluoxetine, pregabalin, amitriptyline, and flupirtine maleate. Herbal medicine interventions included Shaoyao Gancao Decoction, modified Shentong Zhuyu Decoction, Naodesheng Pill, Zhongfeng Huichun Tablet, Jiawei Shentong Zhuyu Decoction, Xingnao Zhitong Decoction, Yiqi Quzhuo Decoction, Huoxue Zhuyu Decoction, Buyang Huanwu Decoction, Xiao Huoluo Dan, Daqinjiao Decoction, MuDan Granule, and modified Huangqi Guizhi Wuwu Decoction. Table 3 provides the detailed composition of the herbal medicine formulas used in the included studies.

**Table 3:** Components of herbal medicine formulations used in the included studies.

Author, year	Preparation name	Dosage form	Main composition (daily dose)	Administration
Xu (19)	Modified Huangqi Guizhi Wuwu Decoction	Decoction	Astragali Radix (*Astragalus membranaceus* (Fisch.) Bunge) (30 g), Paeoniae Radix Alba (*Paeonia lactiflora* Pall.) (30 g), Rehmanniae Radix (*Rehmannia glutinosa* (Gaertn.) DC.) (30 g), Carthami Flos (*Carthamus tinctorius* L.) (10 g), Chuanxiong Rhizoma (*Ligusticum chuanxiong* Hort.) (10 g), Chaenomelis Fructus (*Chaenomeles speciosa* (Sweet) Nakai) (10 g), Zingiberis Rhizoma Recens (*Zingiber officinale* Roscoe) (10 g), Cinnamomi Ramulus (*Cinnamomum cassia* (L.) J. Presl) (9 g), Scorpio (*Mesobuthus martensii* Karsch) (6 g), Jujubae Fructus (*Ziziphus jujuba* Mill.) (4 pieces)	One dose/d, three times daily
Gong and Chen (20)	Jiawei Shentong Zhuyu Decoction	Decoction	Persicae Semen (*Prunus persica* (L.) Batsch) (15 g), Carthami Flos (*Carthamus tinctorius* L.) (15 g), Chuanxiong Rhizoma (*Ligusticum chuanxiong* Hort.) (15 g), Angelicae Sinensis Radix (*Angelica sinensis* (Oliv.) Diels) (15 g), Trogopterori Faeces (*Trogopterus xanthipes* Milne-Edwards) (15 g), Myrrha (*Commiphora myrrha* (Nees) Engl.) (15 g), Pheretima (*Pheretima aspergillum* (E. Perrier)) (10 g), Cyperi Rhizoma (*Cyperus rotundus* L.) (10 g), Cyathulae Radix (*Cyathula officinalis* K. C. Kuan) (15 g), Gentianae Macrophyllae Radix (*Gentiana macrophylla* Pall.) (15 g), Notopterygii Rhizoma (*Notopterygium incisum* Ting ex H. T. Chang) (10 g), Corydalis Rhizoma (*Corydalis yanhusuo* W. T. Wang) (15 g), Spatholobi Caulis (*Spatholobus suberectus* Dunn) (15 g)	One dose/d
Guo et al. (21)	Shaoyao Gancao Decoction	Decoction	Paeoniae Radix Alba (*Paeonia lactiflora* Pall.) (30 g), Glycyrrhizae Radix Praeparata (*Glycyrrhiza uralensis* Fisch. ex DC.) (15 g)	Three times daily
Zhang (18)	Xiao Huoluo Dan	Decoction	Aconiti Radix Cocta (*Aconitum carmichaelii* Debx.) (processed, 9 g), Aconiti Kusnezoffii Radix Cocta (*Aconitum kusnezoffii* Rchb.) (processed, 6 g), Arisaema cum Bile (*Arisaema erubescens* (Wall.) Schott) (bile-processed, 6 g), Pheretima (*Pheretima aspergillum* (E. Perrier)) (10 g), Olibanum (*Boswellia sacra* Flueck.) (10 g), Myrrha (*Commiphora myrrha* (Nees) Engl.) (10 g), Glycyrrhizae Radix (*Glycyrrhiza uralensis* Fisch. ex DC.) (30 g), Mel (Honey) (30 g)	150 mL/time, twice daily
Bi et al. (22)	Modified Shentong Zhuyu Decoction	Decoction	Gentianae Macrophyllae Radix (*Gentiana macrophylla Pall.*) (3 g), Notopterygii Rhizoma (*Notopterygium incisum Ting ex H. T. Chang*) (3 g), Cyperi Rhizoma (*Cyperus rotundus L*.) (3 g), Chuanxiong Rhizoma (*Ligusticum chuanxiong Hort*.) (6 g), Myrrha (*Commiphora myrrha (Nees) Engl.*) (6 g), Trogopterori Faeces (*Trogopterus xanthipes Milne-Edwards*) (6 g), Pheretima (*Pheretima aspergillum (E. Perrier)*) (6 g), Glycyrrhizae Radix (*Glycyrrhiza uralensis Fisch. ex DC*.) (6 g), Persicae Semen *(Prunus persica (L.) Batsch)* (9 g), Carthami Flos (*Carthamus tinctorius L*.) (9 g), Corydalis Rhizoma (*Corydalis yanhusuo W. T. Wang*) (10 g), Spatholobi Caulis (*Spatholobus suberectus Dunn*) (10 g), Angelicae Sinensis Radix (*Angelica sinensis (Oliv.) Diels*) (10 g), Cyathulae Radix (*Cyathula officinalis K. C. Kuan*) (10 g)	400 mL/d, twice daily
Qu (23)	Huoxue Zhuyu Decoction	Decoction	Carthami Flos (*Carthamus tinctorius L.*) (15 g), Persicae Semen (*Prunus persica (L.) Batsch*) (15 g), Chuanxiong Rhizoma (*Ligusticum chuanxiong Hort*.) (15 g), Gentianae Macrophyllae Radix (*Gentiana macrophylla Pall*.) (15 g), Angelicae Sinensis Radix (*Angelica sinensis (Oliv.) Diels*) (15 g), Pheretima (Pheretima aspergillum (*E. Perrier*)) (10 g), Myrrha (*Commiphora myrrha (Nees) Engl.*) (15 g), Cyperi Rhizoma (*Cyperus rotundus L.*) (10 g), Cyathulae Radix (*Cyathula officinalis K. C. Kuan*) (15 g), Notopterygii Rhizoma (*Notopterygium incisum Ting ex H. T. Chang*) (15 g), Corydalis Rhizoma (*Corydalis yanhusuo W. T. Wang*) (15 g), Spatholobi Caulis (*Spatholobus suberectus Dunn*) (15 g)	One dose/d, twice daily
Tang et al. (24)	Buyang Huanwu Decoction	Decoction	Astragali Radix (*Astragalus membranaceus* (Fisch.) Bunge) (120 g), Angelicae Sinensis Radix (*Angelica sinensis* (Oliv.) Diels) (12 g, tail), Paeoniae Radix Rubra (*Paeonia veitchii* Lynch) (10 g), Pheretima (*Pheretima aspergillum* (E. Perrier)) (6 g), Chuanxiong Rhizoma (*Ligusticum chuanxiong* Hort.) (10 g), Carthami Flos (*Carthamus tinctorius* L.) (10 g), Persicae Semen (*Prunus persica* (L.) Batsch) (10 g)	200 mL/d, twice daily
Li (16)	Daqinjiao Decoction	Decoction	Gentianae Macrophyllae Radix (*Gentiana macrophylla* Pall.) (18 g), Glycyrrhizae Radix (*Glycyrrhiza uralensis* Fisch. ex DC.) (12 g), Chuanxiong Rhizoma (*Ligusticum chuanxiong* Hort.) (12 g), Angelicae Sinensis Radix (*Angelica sinensis* (Oliv.) Diels) (12 g), Paeoniae Radix Alba (*Paeonia lactiflora* Pall.) (12 g), Asari Radix (*Asarum heterotropoides* var. *mandshuricum* (Maxim.) Kitag.) (3 g), Notopterygii Rhizoma (*Notopterygium incisum* Ting ex H. T. Chang) (6 g), Saposhnikoviae Radix (*Saposhnikovia di*var*icata* (Turcz.) Schischk.) (6 g), Scutellariae Radix (*Scutellaria baicalensis* Georgi) (6 g), Gypsum Fibrosum (Calcium) (12 g), Angelicae Dahuricae Radix (*Angelica dahurica* (Hoffm.) Benth. & Hook.f. ex Franch. & Sav.) (6 g), Atractylodis Macrocephalae Rhizoma (*Atractylodes macrocephala* Koidz.) (6 g), Rehmanniae Radix (*Rehmannia glutinosa* (Gaertn.) DC.) (6 g), Rehmanniae Radix Praeparata (*Rehmannia glutinosa* (Gaertn.) DC.) (6 g), Poria (*Poria cocos* (Schw.) Wolf) (6 g), Angelicae Pubescentis Radix (*Angelica pubescens* Maxim.) (12 g)	200 mL/time, twice daily
Shen (17)	MuDan Granule	Granule	Astragali Radix (*Astragalus membranaceus* (Fisch.) Bunge), Corydalis Rhizoma (*Corydalis yanhusuo* W. T. Wang), Notoginseng Radix (*Panax notoginseng* (Burk.) F. H. Chen), Paeoniae Radix Rubra (*Paeonia veitchii* Lynch), Chuanxiong Rhizoma (*Ligusticum chuanxiong* Hort.), Salviae Miltiorrhizae Radix (*Salvia miltiorrhiza* Bunge), Carthami Flos (*Carthamus tinctorius* L.), Sappan Lignum (*Caesalpinia sappan* L.), Spatholobi Caulis (*Spatholobus suberectus* Dunn)	One dose/d, three times daily
Zeng and Wang (25)	Naodesheng Pill	Pill	Notoginseng Radix (*Panax notoginseng* (Burk.) F. H. Chen), Chuanxiong Rhizoma (*Ligusticum chuanxiong* Hort.), Carthami Flos (*Carthamus tinctorius* L.), Crataegi Fructus (*Crataegus pinnatifida* Bunge), Puerariae Lobatae Radix (*Pueraria montana var. lobata* (Willd.) Sanjappa & Pradeep)	2 g/time, three times daily
Liang and Ding (26)	Modified Shentong Zhuyu Decoction	Decoction	Gentianae Macrophyllae Radix (*Gentiana macrophylla* Pall.) (3 g), Notopterygii Rhizoma (*Notopterygium incisum* Ting ex H. T. Chang) (3 g), Cyperi Rhizoma (*Cyperus rotundus* L.) (3 g), Chuanxiong Rhizoma (*Ligusticum chuanxiong* Hort.) (6 g), Myrrha (*Commiphora myrrha* (Nees) Engl.) (6 g), Trogopterori Faeces (*Trogopterus xanthipes* Milne-Edwards) (6 g), Pheretima (*Pheretima aspergillum* (E. Perrier)) (6 g), Glycyrrhizae Radix (*Glycyrrhiza uralensis* Fisch. ex DC.) (6 g), Persicae Semen (*Prunus persica* (L.) Batsch) (9 g), Carthami Flos (*Carthamus tinctorius* L.) (9 g), Corydalis Rhizoma (*Corydalis yanhusuo* W. T. Wang) (10 g), Spatholobi Caulis (*Spatholobus suberectus* Dunn) (10 g), Angelicae Sinensis Radix (*Angelica sinensis* (Oliv.) Diels) (10 g), Cyathulae Radix (*Cyathula officinalis* K. C. Kuan) (10 g)	400 mL/d, twice daily
Liu (27)	Xingnao Zhitong Decoction	Decoction	Codonopsis Radix (*Codonopsis pilosula* (Franch.) Nannf.) (15 g), Salviae Miltiorrhizae Radix (*Salvia miltiorrhiza* Bunge) (15 g), Astragali Radix (*Astragalus membranaceus* (Fisch.) Bunge) (20 g), Puerariae Lobatae Radix (*Pueraria montana* var. *lobata* (Willd.) Sanjappa & Pradeep) (30 g), Acori Tatarinowii Rhizoma (*Acorus tatarinowii* Schott) (10 g), Aucklandiae Radix (*Aucklandia costus* Falc.) (6 g)	One dose/d, three times daily
Li et al. (28)	Zhongfeng Huichun Tablet	Tablet	Angelicae Sinensis Radix (*Angelica sinensis* (Oliv.) Diels) (processed with wine), Chuanxiong Rhizoma (*Ligusticum chuanxiong* Hort.), Carthami Flos (*Carthamus tinctorius* L.), Persicae Semen (*Prunus persica* (L.) Batsch), Salviae Miltiorrhizae Radix (*Salvia miltiorrhiza* Bunge), Spatholobi Caulis (*Spatholobus suberectus* Dunn), Lonicerae Caulis (*Lonicera japonica* Thunb.), Trachelospermi Caulis (*Trachelospermum jasminoides* (Lindl.) Lem.), Pheretima (*Pheretima aspergillum* (E. Perrier)), Eupolyphaga (*Eupolyphaga sinensis* Walker), Lycopodii Herba (*Lycopodium japonicum* Thunb.), Cyathulae Radix (*Cyathula officinalis* K. C. Kuan), Scolopendra (*Scolopendra subspinipes mutilans* L. Koch), Leonuri Fructus (*Leonurus japonicus* Houtt.) (parched), Scorpio (*Mesobuthus martensii* Karsch), Clematidis Radix (*Clematis chinensis* Osbeck), Bombyx Batryticatus (*Bombyx mori* Linnaeus) (parched), Chaenomelis Fructus (*Chaenomeles speciosa* (Sweet) Nakai), Bungarus Parvus (*Bungarus multicinctus* Blyth)	four slices/time, three times daily
Dai and Qu (29)	modified Shentong Zhuyu Decoction	Decoction	Gentianae Macrophyllae Radix (*Gentiana macrophylla Pall.*) (3 g), Notopterygii Rhizoma (*Notopterygium incisum Ting ex H. T. Chang*) (3 g), Cyperi Rhizoma (*Cyperus rotundus L*.) (3 g), Chuanxiong Rhizoma (*Ligusticum chuanxiong Hort*.) (6 g), Myrrha (*Commiphora myrrha (Nees) Engl*.) (6 g), Trogopterori Faeces (*Trogopterus xanthipes Milne-Edwards*) (6 g), Pheretima (*Pheretima aspergillum (E. Perrier*)) (6 g), Glycyrrhizae Radix (*Glycyrrhiza uralensis Fisch. ex DC*.) (6 g), Persicae Semen (*Prunus persica (L.) Batsch*) (9 g), Carthami Flos (*Carthamus tinctorius L*.) (9 g), Corydalis Rhizoma (*Corydalis yanhusuo W. T. Wang*) (10 g), Spatholobi Caulis (*Spatholobus suberectus Dunn*) (10 g), Angelicae Sinensis Radix (*Angelica sinensis* (*Oliv.*) *Diels*) (10 g), Cyathulae Radix (*Cyathula officinalis K. C. Kuan*) (10 g)	400 mL/d, twice daily
Peng et al. (30)	Yiqi Quzhuo Decoction	Decoction	Hirudo (*Whitmania pigra* Whitman) (10 g), Scorpio (*Mesobuthus martensii* Karsch) (10 g), Pheretima (*Pheretima aspergillum* (E. Perrier)) (10 g), Cyperi Rhizoma (*Cyperus rotundus* L.) (10 g), Notopterygii Rhizoma (*Notopterygium incisum* Ting ex H. T. Chang) (10 g), Crataegi Fructus (*Crataegus pinnatifida* Bunge) (charred, 15 g), Angelicae Sinensis Radix (*Angelica sinensis* (Oliv.) Diels) (10 g), Astragali Radix (*Astragalus membranaceus* (Fisch.) Bunge) (15 g), Atractylodis Rhizoma (*Atractylodes lancea* (Thunb.) DC.) (10 g), Phellodendri Cortex (*Phellodendron amurense* Rupr.) (10 g), Persicae Semen (*Prunus persica* (L.) Batsch) (10 g), Carthami Flos (*Carthamus tinctorius* L.) (10 g), Glycyrrhizae Radix (*Glycyrrhiza uralensis* Fisch. ex DC.) (6 g)	One dose/d, twice daily
Zhang et al. (31)	Xingnao Zhitong Decoction	Decoction	Codonopsis Radix (*Codonopsis pilosula* (*Franch*.) Nannf.) (15 g), Salviae Miltiorrhizae Radix (*Salvia miltiorrhiza Bunge*) (15 g), Astragali Radix (*Astragalus membranaceus* (*Fisch.*) *Bunge*) (20 g), Puerariae Lobatae Radix (*Pueraria montana var. lobata* (*Willd.*) *Sanjappa & Pradeep*) (30 g), Acori Tatarinowii Rhizoma (*Acorus tatarinowii Schott*) (10 g), Aucklandiae Radix (*Aucklandia costus Falc*.) (6 g)	One dose/d, three times daily

### 3.2 Quality assessment

**Figure 2:**
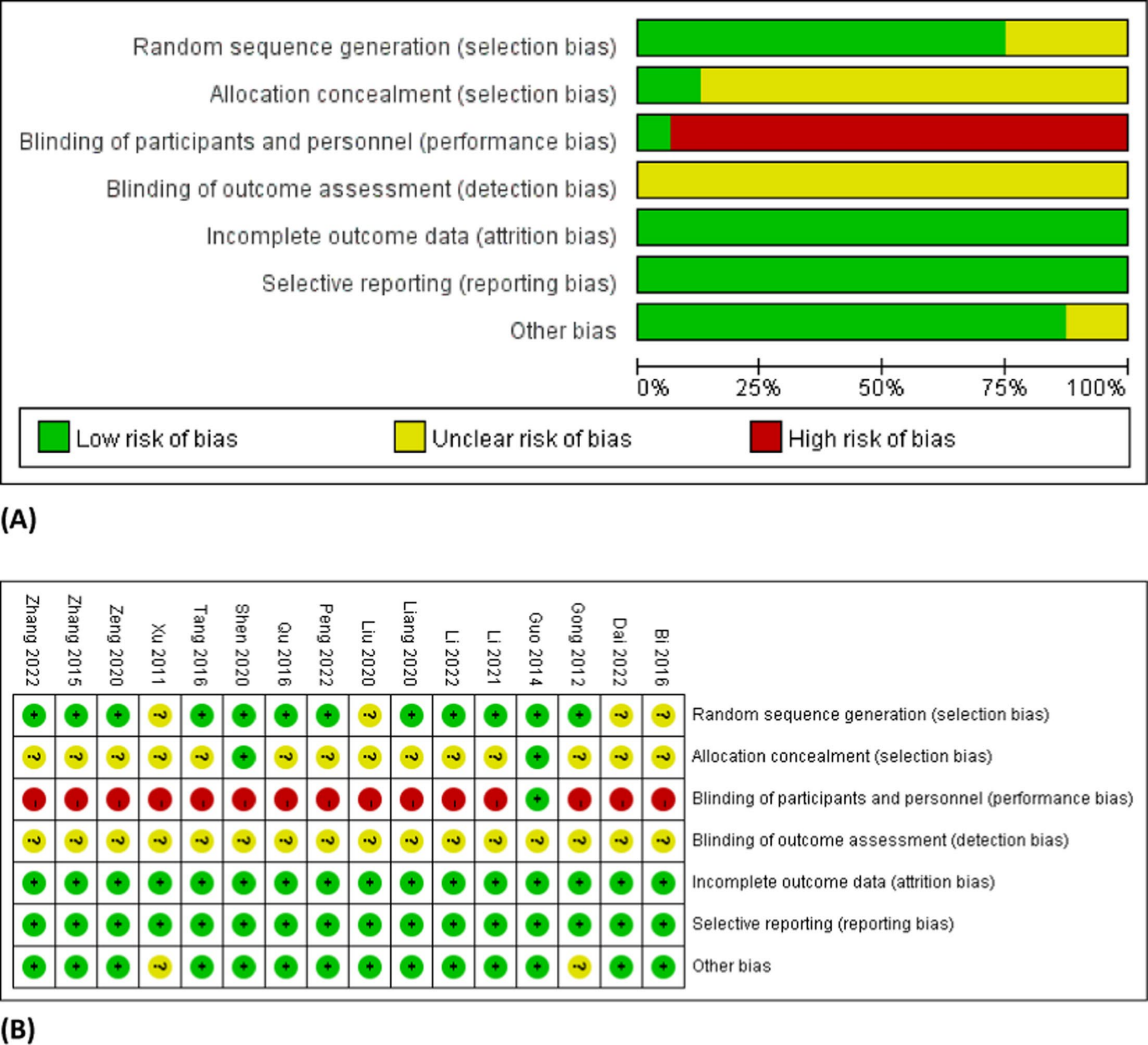
Risk-of-bias summary **(A)** and graph **(B)** were summarized for all included studies.

All 16 randomized controlled trials (RCTs) reported baseline comparability between the experimental and control groups. Although randomization was mentioned in all studies, several (19, 22, 27, 29) did not specify the exact methods used. Allocation concealment procedures were not addressed in any of the reviewed studies, indicating a potential gap in methodological rigor. Regarding blinding, only one study (21) employed a double-blind methodology, while the remaining studies did not implement blinding. Loss to follow-up and withdrawals were mentioned in 4 studies (17, 21, 24, 30), whereas in 12 studies, no cases of loss to follow-up were documented. Figure 2 displays the evaluation of the extent of bias across selected research.

### 3.3 Outcome measures

#### 3.3.1 Pain level rating

##### 3.3.1.1 VAS

**Figure 3:**
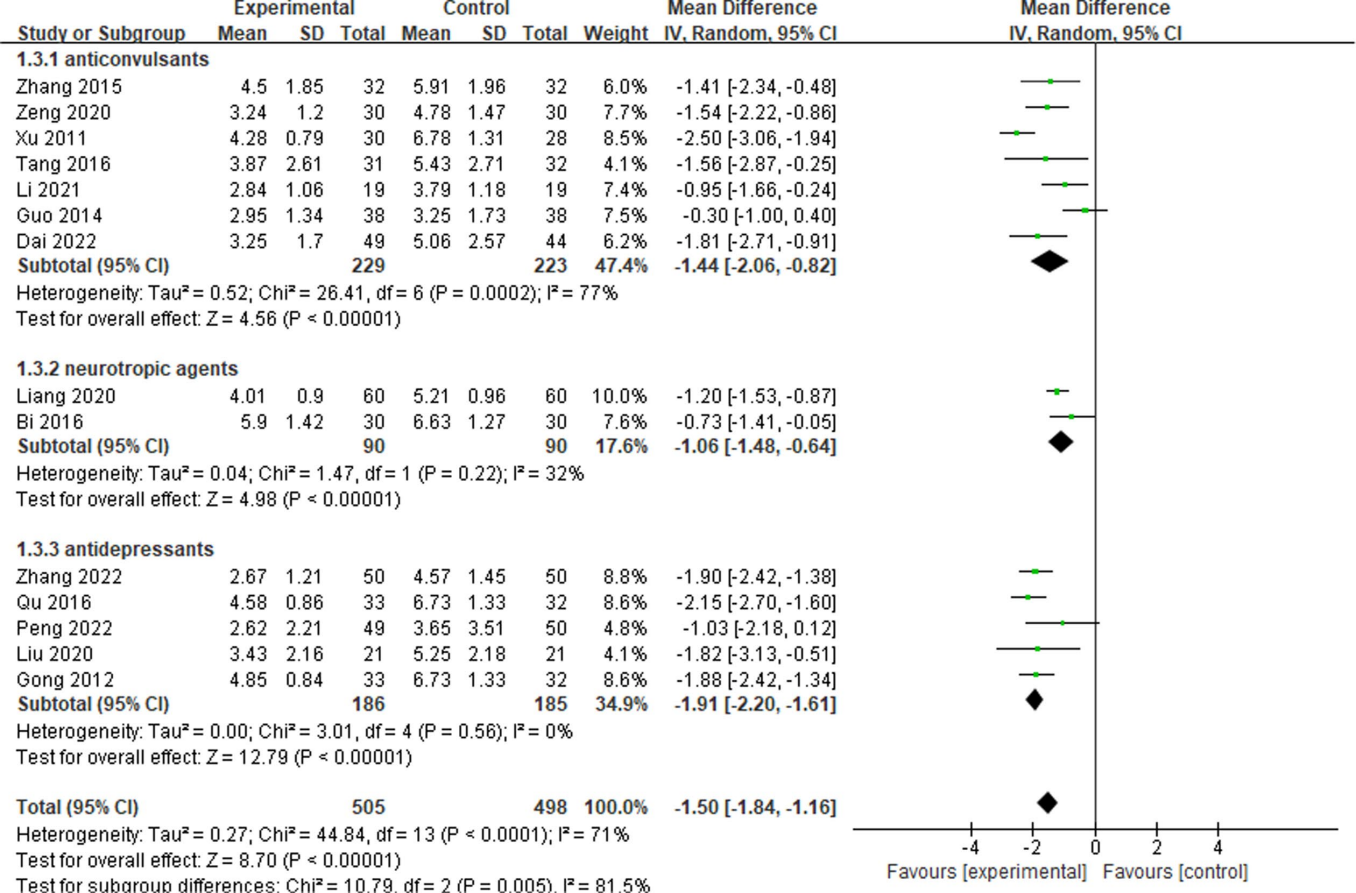
The forest plot of the VAS score based on the type of control drug.

Fourteen studies (18–31) included in the meta-analysis reported VAS scores. Given marked statistical heterogeneity across groups (*p* < 0.0001, *I^2^* = 71%), the analysis was performed using a random-effects model. The pooled results showed that herbal medicine combined with WM significantly reduced VAS scores compared with WM alone (MD = -1.50, 95% CI: −1.84 – -1.16, *p* < 0.0001; Figure 3), suggesting superior analgesic effects of the combination therapy.

To explore potential sources of heterogeneity, subgroup analyses were performed according to the type of WM, including anticonvulsants, neurotropic agents, and antidepressants. Significant reductions in VAS scores were observed across all subgroups: anticonvulsants (MD = −1.44, 95% CI: −2.06 – -0.82, *I^2^* = 77%), neurotropic agents (MD = −1.06, 95% CI: −1.48 – -0.64, *I^2^* = 32%), and antidepressants (MD = −1.91, 95% CI: −2.20 – -1.61, *I^2^* = 0%). The test for subgroup differences was statistically significant (*p* = 0.005), indicating that the type of WM may partially contribute to the observed heterogeneity. To assess robustness, sensitivity analyses were carried out by sequentially removing each trial, confirming the stability of the overall effect size.

##### 3.3.1.2 PPI

**Figure 4:**
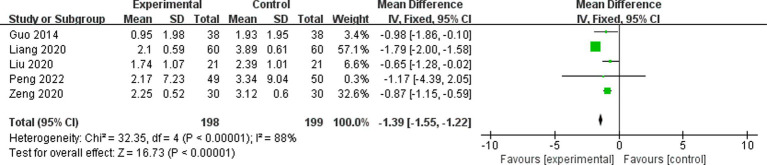
The forest plot of the PPI score.

PPI scores were presented in five studies (21, 25–27, 30), initially showing high heterogeneity (*p* < 0.00001, *I^2^* = 88% > 50%). Using a random-effects model, the pooled results showed that herbal medicine combined with WM significantly reduced PPI scores compared with WM alone (MD = −1.39, 95% CI: −1.55 – -1.22, *p* < 0.00001; Figure 4).

##### 3.3.1.3 PRI

**Figure 5:**
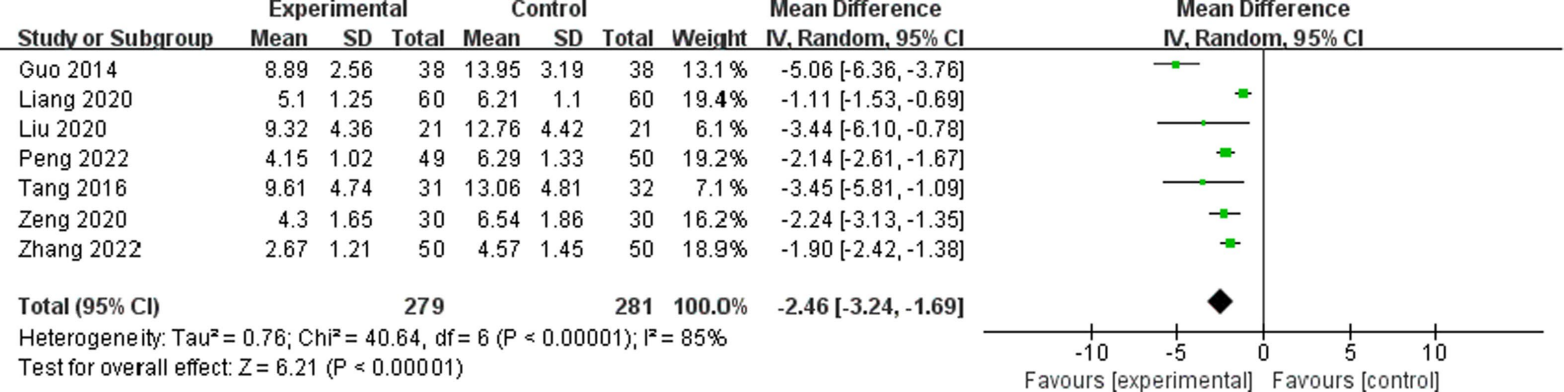
The forest plot of the PRI score.

The PRI score, reported in seven studies (21, 24–27, 30, 31), exhibited substantial heterogeneity post-treatment (*p* < 0.00001, *I^2^* = 88% > 50%). Therefore, analyses were performed under a random-effects model, revealing a significant reduction in PRI scores favoring participants in the therapy arm (MD = −2.46, 95% CI: −3.24 – -1.69, *p* < 0.00001; Figure 5). The data imply that the treatment group achieved more pronounced pain relief relative to the control group.

#### 3.3.2 Evaluation of anxious state

##### 3.3.2.1 HAMA

**Figure 6:**
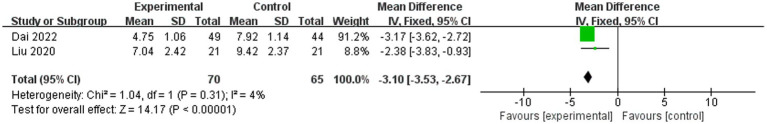
The forest plot of the HAMA score.

Two studies (27, 29) using HAMA revealed that herbal medicine combined with WM therapy significantly reduced anxiety scores compared with WM alone (MD = −3.10, 95% CI: −3.53 – -2.67, *p* < 0.00001; Figure 6). These findings indicate that herbal medicine supplementation is effective in alleviating anxiety symptoms beyond the benefits of WM as monotherapy.

##### 3.3.2.2 SAS

**Figure 7:**
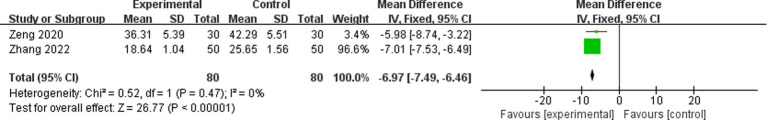
The forest plot of the SAS score.

According to a meta-analysis of studies (25, 31) using SAS, anxiety levels were significantly lower in patients receiving combined herbal medicine and WM therapy than in patients receiving WM alone (MD = −6.97, 95% CI: −7.49 – -6.46, *p* < 0.00001; Figure 7).

#### 3.3.3 Evaluation of depressive state

##### 3.3.3.1 HAMD

**Figure 8:**
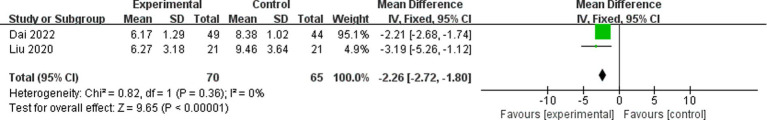
The forest plot of the HAMD score.

Similarly, studies (27, 29) employing HAMD showed a significant decrease in depression scores with combined herbal medicine and WM therapy in contrast to WM alone (MD = −2.26, 95% CI: −2.72 – -1.80, *p* < 0.00001; Figure 8). These suggest that the addition of herbal medicine to WM leads to substantial improvements in depressive outcomes for individuals with PSTP.

##### 3.3.3.2 SDS

**Figure 9:**
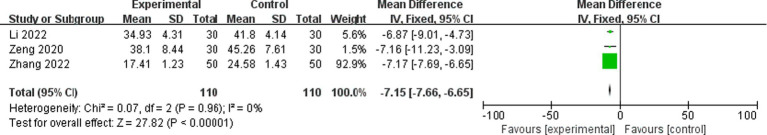
The forest plot of the SDS score.

Meta-analysis of three studies (16, 25, 31) using SDS revealed a significant reduction in depressive symptoms among patients receiving combined herbal medicine and WM therapy compared to those receiving WM solely (MD = −7.15, 95% CI: −7.66 – -6.65, *p* < 0.00001; Figure 9). Sensitivity analysis showed that exclusion of master’s thesis studies (16) did not materially change the pooled results, indicating robust findings.

#### 3.3.4 Total effective rate

**Figure 10:**
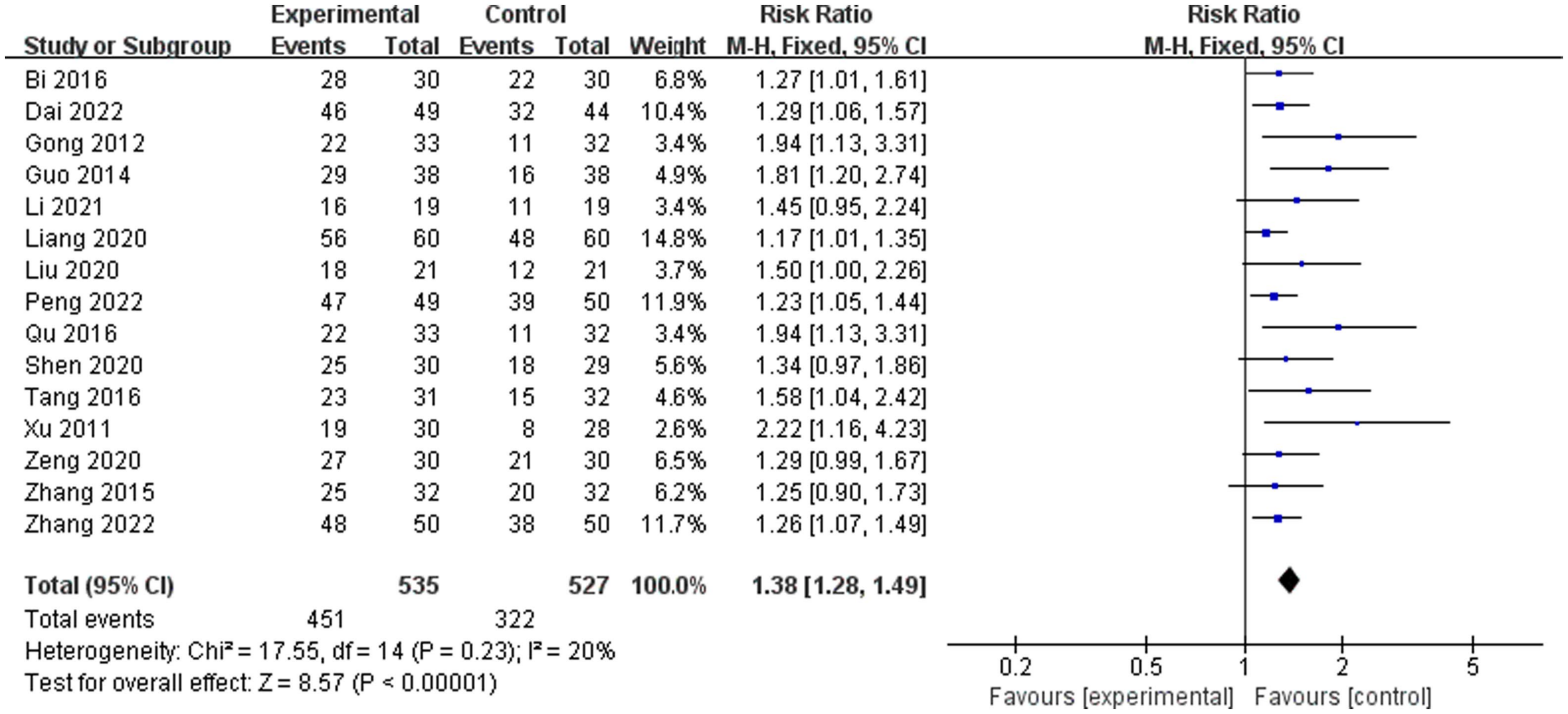
The forest plot of the total effective rate.

The meta-analysis encompassed 15 studies (17–31) with 1,062 patients, of whom 535 received the experimental treatment and 527 were in the control arm. Given the low heterogeneity among studies (*p* = 0.23 > 0.1, *I^2^* = 20 < 50%), the meta-analysis was performed using a common-effect model. The total effective rate showed a statistically significant difference between the two groups (RR = 1.38, 95% CI: 1.28–1.49, *p* < 0.00001; Figure 10). Sensitivity analysis confirmed the robustness of the results after excluding master’s thesis studies (17, 18).

#### 3.3.5 Adverse reactions

**Figure 11:**
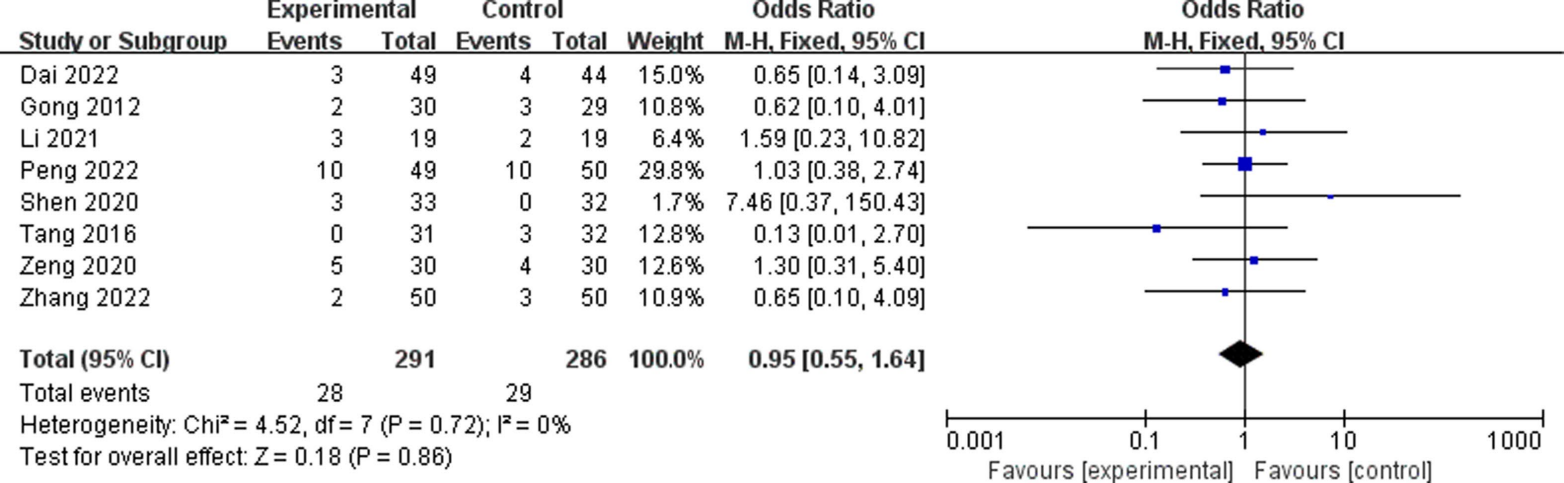
The forest plot of the adverse event rate.

Eight studies (17, 23–25, 28–31) examined adverse reactions during treatment, documenting incidences such as nausea, abdominal pain, diarrhea, dizziness, drowsiness, rash, and occasional abnormalities in liver and kidney function. Notably, no severe side effects were reported across the studies. The follow-up durations across studies were relatively short, predominantly ranging from 2 to 8 weeks. Our analysis did not find any significant differences in adverse effect rates between participants treated with combined herbal medicine and WM and those receiving WM monotherapy (RR = 0.95, 95% CI: 0.55–1.64, *p* = 0.86; Figure 11). Sensitivity analysis confirmed the robustness of the results after excluding master’s thesis study (17).

### 3.4 Publication bias detection

**Figure 12:**
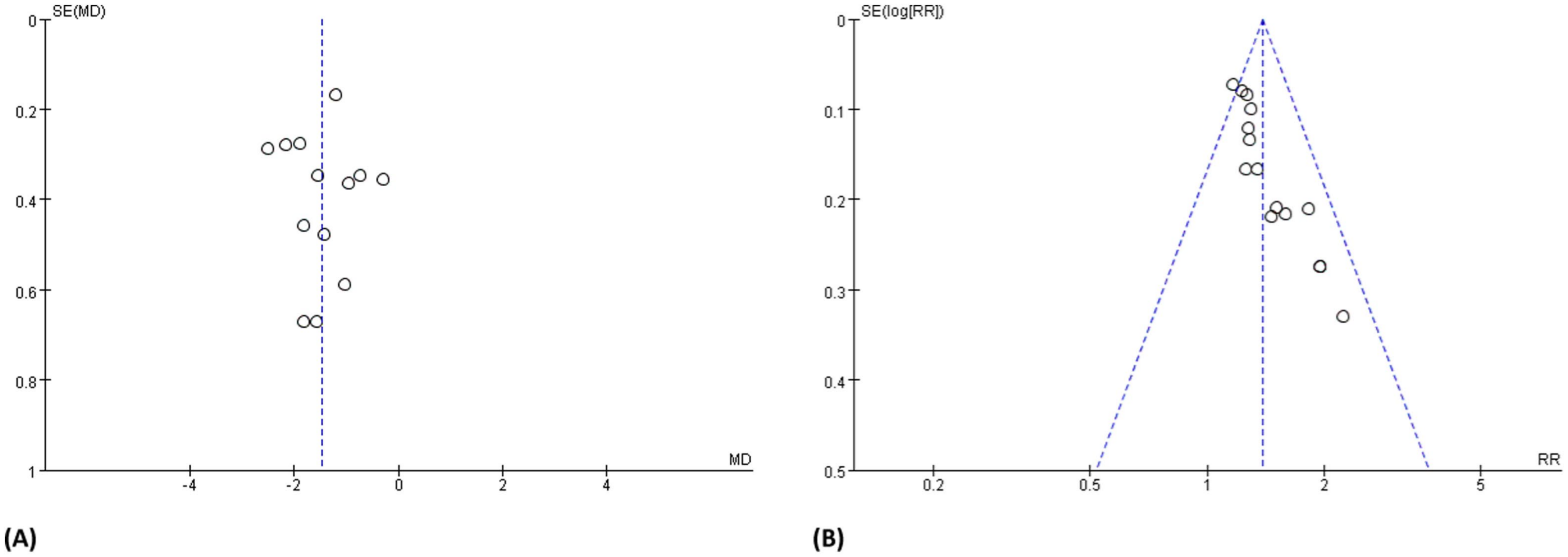
Funnel plot for the VAS **(A)** and total effective rate **(B)**.

**Figure 13:**
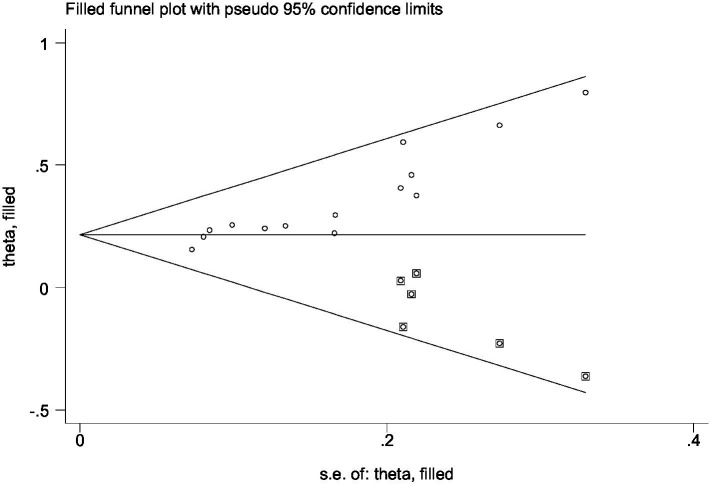
Funnel plot for total effective rate adjusted by the trim-and-fill method.

Funnel plots were generated for VAS scores and total effective rate, and Egger’s test was performed to further evaluate funnel plot asymmetry. Mild asymmetry was observed in the funnel plot for VAS scores, but it did not reach statistical significance according to Egger’s test (*p* = 0.14), implying that publication bias was minimal (Figure 12). In contrast, significant asymmetry was identified in funnel plot of the total effective rate. With a significant Egger’s test (*p* < 0.001), the presence of publication bias was acknowledged (Figure 12), which may result from the underreporting of studies with negative or nonsignificant results. To account for this bias, the trim-and-fill approach was utilized, estimating the existence of approximately seven missing studies (Figure 13). After imputation of these theoretical studies, the adjusted pooled risk ratio retained its statistical significance (RR = 1.242, 95% CI: 1.168–1.320).

### 3.5 GRADE assessment

We next evaluated the overall quality of evidence with the GRADE profiler, followed by a trial sequential analysis to reinforce our conclusions. The certainty of evidence regarding the overall efficacy rate, VAS score, PPI, and PRI score as outcome markers was assessed using the GRADE profiler. The certainty of evidence was deemed to be very low for PRI, low for VAS score and total effective rate, and moderate for PPI (Table 4). These ratings were primarily due to several factors that downgraded the quality of evidence across studies, including inadequate methodology, small sample sizes, significant heterogeneity among studies, and potential publication bias. Despite these limitations, the findings from the meta-analysis provide preliminary evidence regarding the potential benefits of combining herbal medicine with WM for PSTP.

**Table 4:** Certainty of the evidence of Chinese medicines combine with Western medicines in the management of post-stroke thalamic pain according to the grading of recommendations, assessment, development, and evaluation (GRADE) method.

Outcomes	No. of studies (participants)	Effect size (95% CI)	Risk of bias	Inconsistency	Indirectness	Imprecision	Publication bias	Quality of evidence
VAS	14 studies (1003)	MD = −1.50 (−1.84, −1.16)	serious^a^	serious^b^	not serious	not serious	not serious	⨁⨁◯ ◯ Low
PPI	5 studies (397)	MD = −1.39 (−1.5, −1.22)	serious^a^	not serious	not serious	not serious	Unclear	⨁⨁⨁ ◯ Moderate
PRI	6 studies (460)	MD = −2.46 (−3.24, −1.69)	serious^a^	serious^b^	not serious	serious^c^	Unclear	⨁◯ ◯ ◯ Very low
Total effective rate	15 studies (1062)	RR = 1.38 (1.28, 1.49)	serious^a^	not serious	not serious	not serious	serious^d^	⨁⨁◯ ◯ Low

^a^Lack of blinding and allocation concealment. ^b^Heterogeneity among included studies (*p* < 0.1 or I2 ≥ 50%). ^c^The sample size included in the outcome is too small. ^d^Publication bias is probably.

### 3.6 TSA

**Figure 14:**
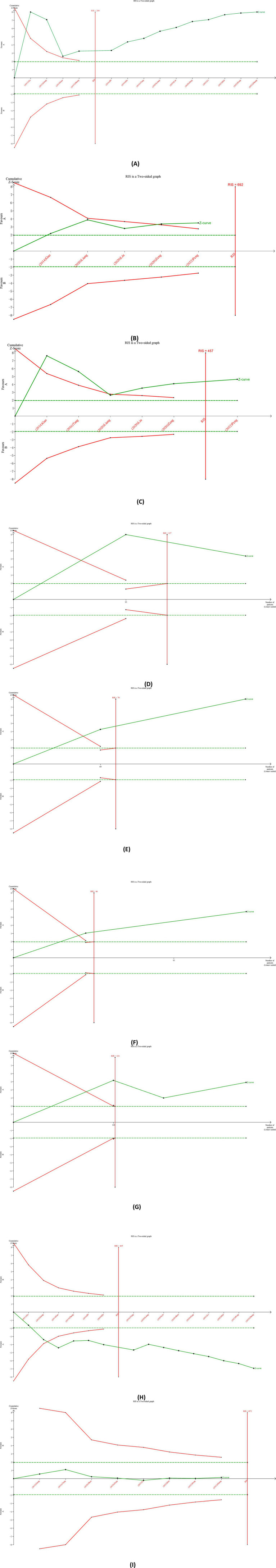
TSA for the VAS **(A)**, PPI **(B)**, PRI **(C)**, HAMA **(D)**, SAS **(E)**, HAMD **(F)**, SDS **(G)**, total effective rate **(H)** and adverse reactions **(I)**.

TSA was conducted on the VAS, PPI, PRI, HAMA, SAS, HAMD, SDS, total effective rate, and rate of adverse reactions to minimize the potential for spurious positive results caused by random error. Figure 14 illustrates that the cumulative Z-curve (green solid line) consistently exceeded both the TSA boundaries (red curve) and the conventional significance thresholds (green dashed line), indicating a robust outcome. Specifically, the cumulative Z-curve for VAS, PRI, HAMA, SAS, HAMD, SDS, total effective rate and rate of adverse reactions, surpassed the required information size (RIS) threshold, suggesting sufficient data to draw reliable conclusions. Although the cumulative patient count for PPI was below the RIS estimate, the TSA boundary was crossed, suggesting that the current evidence may be sufficient for this outcome. These TSA results provide additional statistical support for the potential benefit of combining herbal medicine with WM in treating PSTP.

## 4 Discussion

### 4.1 Summary of main findings

Based on 16 RCTs including 1,122 participants, the meta-analysis suggested that adjunctive herbal medicine may have potential benefits in the treatment of PSTP. The adjunctive therapy exhibited substantial efficacy in reducing pain without significantly increasing adverse events. By using the TSA, we were able to show that the main outcome indicators (the total effective rate, VAS, and PRI score) obtained the RIS, and the cumulative Z-curve consistently reached beyond the trial sequential monitoring boundary (TSMB). For the PPI outcome, the cumulative Z-curve demonstrated a notable early breach of the TSMB prior to reaching the RIS. These findings provide further support for the potential benefit of combining herbal medicine with WM in managing PSTP. GRADE assessment classified the evidence as low for total effective rate and VAS, very low for PRI, and moderate for PPI scores. Owing to methodological limitations in the included studies, these findings should be interpreted with caution.

### 4.2 Clinical and scientific implications

Pharmacological management of PSTP remains limited. Tricyclic antidepressants (e.g., amitriptyline) are commonly prescribed but can cause side effects, including hypotension and arrhythmias (32). Serotonin-norepinephrine reuptake inhibitors, including duloxetine and venlafaxine (33), along with gabapentinoids provide alternative options but often require combination therapy due to modest efficacy. Investigations have also focused on non-pharmacological interventions, including rTMS, invasive motor cortex stimulation (34–37), and acupuncture (38–40), underscoring the need for multimodal treatment strategies.

Our meta-analysis indicates that combining herbal medicine with WM may provide additional benefits in improving clinical response (total effective rate), reducing pain intensity (VAS, PPI, and PRI) and alleviating anxiety and depression in patients with PSTP, with a safety profile comparable to WM therapy alone. The total effective rate was frequently reported across included studies as a composite measure of clinical response. However, it is not a standardized outcome in international pain research. Therefore, these findings should be interpreted alongside validated pain-related scales, including VAS, PRI, and PPI, which provide more robust and comparable measures of pain intensity. From a clinical perspective, these findings may be relevant, as mood disorders are common comorbidities in PSTP and may further contribute to disease burden (41). For clinicians, an integrative approach could be considered as a potential adjunct to standard pharmacological treatments (e.g., gabapentinoids or antidepressants), although it’s routine clinical application requires further confirmation.

The minimal clinically important difference (MCID) is a widely used indicator for interpreting the clinical relevance of treatment effects. Although MCID values for chronic pain have been reported in previous studies, no established MCID specific to post-stroke thalamic pain currently exists (42). Therefore, evidence from related chronic pain conditions suggests that the MCID for VAS is approximately 1.5–2.0 points (43). In the present study, baseline VAS scores ranged from 3.25 to 6.78, indicating moderate pain severity, which may influence the interpretation of MCID thresholds. The pooled mean difference of −1.50 suggests a clinically meaningful trend of pain reduction, although the exact clinical significance in PSTP remains uncertain. Future studies should establish condition-specific and baseline-adjusted MCID thresholds for PSTP to better interpret treatment effects.

Among the herbal interventions examined, Shentong Zhuyu Decoction was the most frequently investigated (*n* = 4), followed by Xingnao Zhitong Decoction (*n* = 2). These prescriptions largely adhere to the therapeutic approach of “activating blood circulation and resolving stasis,” which is consistent with the traditional Chinese medicine theory that blood stasis plays an important role in the pathogenesis of pain symptoms (44). The potential benefits of herbal medicine may be explained by its multi-component and multi-target pharmacological actions. Blood-activating herbs such as Honghua (*Carthami Flos*) and Danshen (*Salvia miltiorrhiza*) improve microcirculation and inhibit platelet aggregation (45). Taking safflower as an example, its key bioactive component hydroxy safflor yellow A demonstrates potential in alleviating thalamic pain by modulating the MAPK/p38/iNOS pathway (46) to reduce nitric oxide release, while also suppressing HPA axis hyperactivity, oxidative stress, and neuroinflammation (47, 48). Evidence from related central post-stroke pain research suggests that the PIK3CG/NLRP3 signaling axis may be involved in the analgesic effects of Danshenol B (49). Meanwhile, anti-inflammatory and neuroprotective herbs such as Chuanxiong (*Ligusticum chuanxiong*) and Danggui (*Angelica sinensis*) modulate the NF-κB/p38 signaling pathways, thereby reducing neuroinflammation and central sensitization (50). In addition, flavonoids and other bioactive compounds from herbal medicine have demonstrated antidepressant and anxiolytic effects by regulating the hypothalamic–pituitary–adrenal axis and neurotransmitter balance (51–53). These mechanisms provide a plausible explanation for the dual improvement in pain and mood symptoms observed in our analysis.

### 4.3 Strengths and limitations

To our knowledge, this is the first systematic review and meta-analysis to evaluate herbal medicine as an adjunct to WM for the management of PSTP, providing an updated evidence-based synthesis of this therapeutic approach. This study has several strengths. We employed TSA to evaluate the main outcome measures. The results demonstrated that the cumulative Z-curves for the total effective rate, VAS, and PRI scores all crossed the trial sequential monitoring boundaries, thereby providing further statistical support for the robustness of the meta-analysis findings and reducing the risk of type I errors. In addition, beyond assessing analgesic efficacy, this study comprehensively evaluated the intervention’s effects on comorbid anxiety and depression. This multidimensional assessment aligns with the common clinical presentation of PSTP, which frequently involves affective disturbances, offering a more holistic view of patient outcomes.

The interpretation of our findings is limited by several factors. First, the methodological quality of the included RCTs was generally low. None of the included studies reported allocation concealment, and only one trial implemented a double-blind design. Most studies were at unclear risk of bias in several domains, including selection, performance, and detection bias. These limitations reduce the overall confidence in the pooled effect estimates and are reflected in the overall low-to-moderate certainty of evidence as assessed using the GRADE framework. In addition, a small proportion of included studies were unpublished master’s theses rather than peer-reviewed journal articles. Although all studies were assessed using standardized risk of bias tools and incorporated into the GRADE framework, the inclusion of gray literature may raise concerns regarding reporting quality. Second, substantial heterogeneity was observed across VAS, PRI, and PPI, likely due to differences in herbal formulations, types of Western medicine, treatment duration, and baseline characteristics. The lack of standardization of herbal prescriptions may also have contributed. Subgroup analyses and meta-regression were not feasible due to limited study numbers. Third, the evaluation of therapeutic efficacy relied largely on subjective scales (VAS, PRI, and PPI). These instruments are susceptible to patients’ psychological and emotional states and lack specificity for neuropathic pain assessment, potentially compromising the accuracy of outcome measurements (54). Future investigations should incorporate more objective measures, for instance, the Leeds Assessment of Neuropathic Symptoms and Signs (LANSS) (55, 56) or electrophysiological measures like quantitative EEG (57). Fourth, the included RCTs had relatively short follow-up durations, which may be insufficient to assess delayed adverse events and long-term safety outcomes. Finally, all included RCTs were conducted in China, which may limit the external validity and generalizability of the findings to other healthcare systems and populations.

## 5 Conclusion

Our findings point to the potential of integrated herbal medicine and WM for improving both pain and depressive symptoms in the management of PSTP. However, the certainty of evidence is limited due to generally low methodological quality of the included studies, particularly the lack of allocation concealment and blinding, which reduces confidence in the pooled estimates. Future well-designed, multicenter RCTs are needed to confirm these findings, with improved methodological rigor, predefined clinically meaningful outcomes, and longer follow-up periods to assess the durability and safety of treatment effects.

## Data Availability

The original contributions presented in the study are included in the article/Supplementary material, further inquiries can be directed to the corresponding author/s.
